# Angiopoietin-like-3 knockout protects against glomerulosclerosis in murine adriamycin-induced nephropathy by attenuating podocyte loss

**DOI:** 10.1186/s12882-019-1383-1

**Published:** 2019-05-24

**Authors:** Rufeng Dai, Haimei Liu, Xinli Han, Junchao Liu, Yihui Zhai, Jia Rao, Qian Shen, Hong Xu

**Affiliations:** 1 Department of Nephrology, Children’s Hospital of Fudan University, Shanghai, 201102 China; 2 Shanghai Kidney Development & Pediatric Kidney Disease Research Center, Shanghai, 201102 China; 3 Department of Rheumatism, Children’s Hospital of Fudan University, Shanghai, 201102 China; 4 Department of Chinese Medicine, Children’s Hospital of Fudan University, Shanghai, 201102 China

**Keywords:** Angiopoietin-like-3 knockout, Glomerulosclerosis, Podocyte loss, Adriamycin-induced nephropathy

## Abstract

**Background:**

Angiopoietin-like-3 (Angptl3) knockout is known for its protective effects on podocyte injury and proteinuria in the early stage of adriamycin (ADR) nephropathy. The current study re-evaluated the renoprotective effect of Angptl3 knockout in chronic ADR nephropathy and attempted to explore the mechanism underlying the effect associated with Angptl3 knockout in glomerulosclerosis.

**Methods:**

B6; 129S5 mice were injected with ADR to induce nephropathy. Kidney structure and serum and urine parameters were observed during long-term follow-up. Cultured primary mouse podocytes were exposed to ADR and analyzed for the expression of some relative proteins. Podocyte loss was analyzed in both in vivo and in vitro experiments.

**Results:**

Angptl3 knockout attenuated proteinuria and hypoproteinemia, protected renal structure and function, and improved the survival of mice over the whole process of ADR nephropathy. Furthermore, Angptl3 knockout reduced the numbers of the detached and apoptotic cells in the renal tissue and alleviated podocyte loss in mice with ADR chronic nephropathy, thereby, delaying the glomerulosclerosis formation. Additional results in vitro showed that Angptl3 knockout attenuated ADR-induced primary podocyte loss, including podocyte detachment and apoptosis.

**Conclusion:**

In addition to serving a renoprotective role in the early stage of ADR nephropathy, Angptl3 knockout contributed to disease amelioration throughout the ADR nephropathy process. Angptl3 knockout effectively delayed glomerulosclerosis formation by attenuating podocyte loss through rescuing podocytes from detachment and apoptosis. Angptl3 antagonists or inhibitors might have therapeutic potential in the occurrence and progression of nephropathy.

**Electronic supplementary material:**

The online version of this article (10.1186/s12882-019-1383-1) contains supplementary material, which is available to authorized users.

## Background

Chronic kidney disease (CKD) is a substantial worldwide burden on patients and society. Pathologically, glomerulosclerosis accounts for the vast majority of CKD cases leading to end-stage renal disease (ESRD), and podocyte loss is closely related to the occurrence and progression of glomerulosclerosis [1–3]. The mechanism of glomerulosclerosis and therapeutic interventions aimed at the prevention or reversion of glomerulosclerosis have been intensively investigated. Despite decades of extensive research, no specific treatments are available to prevent or reverse glomerulosclerosis.

Angiopoietin-like protein 3 (Angptl3) is a secreted protein that is mainly produced by the liver and minimally expressed in the normal kidneys [4]. Angptl3 plays important roles in the regulation of lipid metabolism [4], angiogenesis [5], the stem cell proliferation process [6], insulin resistance [7], hepatocellular carcinoma [8] and some other biological functions [9–11]. Our previous work revealed increased Angptl3 expression in the glomeruli of children with nephrotic syndrome (including minimal change disease and glomerulosclerosis) and animal models of Adriamycin (ADR) nephropathy, and in ADR- or puromycin aminonucleoside (PAN)- treated cultured podocytes [12–16]. Moreover, we found that Angptl3 overexpression stimulates podocyte F-actin rearrangement in vitro [17], increases podocyte motility [16] and accelerates podocyte loss (including podocyte detachment and apoptosis) [18], which may be related to promoting proteinuria. To further clarify the role of altered Angptl3 expression as a regulatory or modulatory factor in renal proteinuria, we used Angptl3 gene knockout mice. Our previous results showed that Angptl3 knockout was associated with renoprotection in the early stage of ADR nephropathy [19]. However, ADR nephropathy usually progresses to end stage kidney disease, which is phenotypically characterized by glomerulosclerosis [20]. Here, we found that Angptl3 knockout not only ameliorates ADR nephropathy in the early stage but also protects against its progression. Angptl3 knockout ameliorated glomerulosclerosis by attenuating podocyte loss via rescuing podocytes detachment and apoptosis. The current study proposes that Angptl3 antagonists or inhibitors are potential and attractive therapeutic candidates for podocyte injury and proteinuria in the occurrence and progression of nephropathy, which has not been proposed in other studies.

## Methods

### Mouse models

All animal experiments were performed in accordance with protocols approved by the Animal Care and Use Committee of the Institute of Developmental Biology and Molecular Medicine (IDMIACUC) at Fudan University. As our previous protocol described [19], Angptl3−/− mice were bred with two Angptl3+/− mice (B6; 129S5-Angptl3tm1Lex, No. 032146-UCD, Mutant Mouse Regional Resource Centers, Davis, CA), the mouse genotypes were identified by polymerase chain reaction (PCR), and female 8-week-old Angptl3+/+ and Angptl3−/− mice received an intravenous ADR (Sigma-Aldrich, St. Louis, USA; 25 mg/kg dissolved in isotonic saline to a final concentration of 5 μg/μL) on day 0. The control mice received an identical volume of saline intravenously. The ADR-injected mice were given two daily intraperitoneal injections of 2 ml of glucose-electrolyte solution at 36 h after the ADR injections from day 2 to day 6, to prevent weight loss due to low appetite [19]. At weeks 1, 2, 4, 6, 8 and 12, spot urine and blood samples were collected for biochemical studies. The mice were sacrificed at the indicated time points, and the kidneys were removed after saline perfusion for histological studies. There were 6 mice in each group at each time point (Additional file 2: Figure S1a). All mice were euthanized via cervical dislocation following the IDMIACUC Animal Protocol.

### Morphometric analysis

Kidneys were re-moved from the euthanized mice. Tissue samples (0.4 cm × 0.3 cm × 0.3 cm) were fixed in 4% paraformaldehyde, embedded in paraffin, sectioned at 4 μm, stained with periodic acid-schiff (PAS), and examined under a light microscope. For the transmission electron microscope (TEM), the renal cortices were cut (1 mm^3^) and fixed in 2.5% glutaraldehyde overnight, post fixed in 1% osmium tetroxide, dehydrated and embedded according to routine procedures.

Images from at least 10 (until glomerulosclerosis was observed, otherwise the whole kidney was sectioned) sequential glomerular cross-sections (4 μm thickness) approximately at the glomerular equator (0.4 cm × 0.3 cm) were collected for each histological section and assessed by a blinded observer. The number of cells in each glomerular Bowman’s capsule cross section was counted as described by Kim YH et al. [21], with a minor modification. Thirty glomeruli from each mouse of the 6 mice per group were assessed via light microscopy, and 1 glomerulus from each mouse of the 6 mice per group was evaluated under a TEM.

### Urine ribonucleic acid preparation and quantitation

Urine was collected overnight (average 12 h), and the total urine pellet ribonucleic acids (RNA) was isolated using the protocol of the RNeasy Mini Kit (Qiagen, Hilden, Germany) [22]. The steady-state amount of nephrin, and podocin messenger ribonucleic acids (mRNAs) was analyzed by real-time PCR using the Stratagene Mx3000p real-Time PCR System (Santa Clara, CA, United States).The analysis was conducted as described in the instructions of the SYBR FAST qPCR Kit (KAPA). RT-qPCR was conducted 3 times in duplicate using each of the cDNA samples. The amplified transcripts were quantitated with the comparative method using glyceraldehyde-phosphate dehydrogenase (GAPDH) as an internal control. The primers were designed using Primer express software (Primer premier 5.0) based on GenBank accession numbers. The sequences used were: Nephrin, 5′- CCC AGG TAC ACA GAG CAC AA-3′ and 5′- CTC ACG CTC ACA ACC TTC AG-3′; Podocin, 5′- TCT CCT GGA AAG GAA GAG CA-3′ and 5′- GTC TTT GTG CCT CAG CTT CC-3′; GAPDH, 5′- TGC GAC TTC AAC AGC AA CTC-3′ and 5′-ATG TAG GCC ATG AGG TCC AC-3′.

### Podocyte culture and treatment

The kidney cortex of mice was removed, minced and subjected to standard differential three-step sieving under sterile conditions to isolate the glomeruli as previously reported [23]. The isolated glomeruli were plated in 10 cm plates coated with type I collagen. The culture medium consisted of K1 medium and NIH 3 T3 medium mixed in a 1:1 ratio as described [24]. The outgrowth of podocytes started between days 3 and 4. After 6 days of primary culture, the glomeruli were removed and the podocytes were trypsinized and replated onto plates coated with type I collagen for further growth, expansion and analysis (Additional file 2: Figure S1b). We used immunoflurescence staining and flow cytometry analysis to identify the purity of the primary podocytes. We confirmed that most (more than 90%) of the cells were podocytes (Additional file 2: Figure S1d and e). We performed a 3-[4, 5] dimethylthiazol-2,5-diphenyltetrazolium bromide (MTT) assay to quantify cell viability, and the assay demonstrated that the podocyte cellular activity of passage 1 day (P1) podocytes increased significantly on day 3, peaked on day 7, and then slowly decreased (Additional file 2: Figure S1c). Therefore, we used P1 podocytes at days 3 to 9 in our in vitro study. Podocytes were treated with ADR (1 μmol/ml, 48 h) to induce podocyte loss, and control podocytes were treated with phosphate buffer solution.

The urine protein excretion assay, serum biochemistry analysis, PAS histostain, transmission electron microscopy, immunofluorescence labeling, MTT assay, detachment assay, apoptosis detection assay and Western blot analysis were described in Additional file 1: Supplementary Methods.

### Statistical analysis

Most analyses and calculations were performed using Prism® version 5 (GraphPad Software, La Jolla, CA, USA) and IBM SPSS Statistics version 19.0 (IBM Corporation, Armonk, NY, USA). Values, which are expressed as the mean ± SEM, were compared by Student’s t-test, one-way analysis of variance or the nonparametric Kruskal-Wallis test, followed by the Student Newman-Keuls posthoc test. Statistical significance was set at *P* < 0.05.

## Results

### Angptl3 knockout plays a certain protective role in the occurrence and progression of mice ADR nephropathy

Angptl3 knockout has previously been shown to play an important role in the early stage of ADR nephropathy [19]. To observe whether the Angptl3 knockout plays a similar renal protective role in the end stage of nephropathy, we compared differences in proteinuria, hypoproteinemia, renal function, general condition (weight, fur quality, activity and appetite) and survival rates among different experimental groups at different time points up to 12 weeks in our ADR nephropathy mouse model. Angptl3 knockout (Angptl3−/−) mice have phenotypes similar to those of wild-type (Angptl3+/+) mice, except for the presence of hypolipidemia [19]. Therefore, we used average data from Angptl3+/+ and Angptl3−/− mice treated with or without saline injection as controls.

**Fig. 1:**
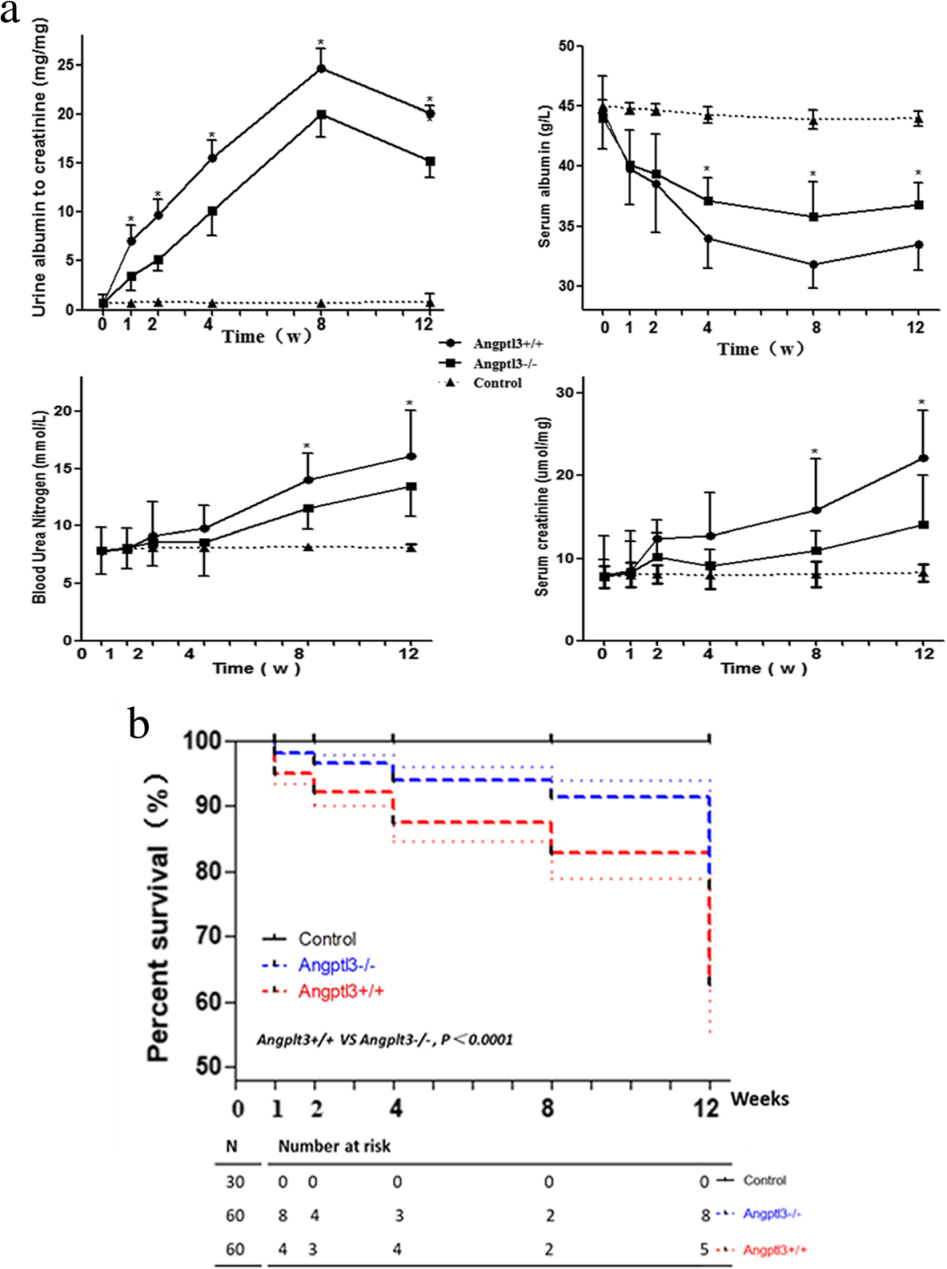
Angptl3 knockout attenuates proteinuria and hypoproteinemia, and improves renal function and survival. **a** Time courses of urine albumin: creatinine, ratio, serum albumin level, blood urea nitrogen level, serum creatinine level and survival percentage **b** of Angptl3+/+ and Angptl3−/− mice with or without ADR injection (each *n* = 6)

Proteinuria was seen as early as 1 week following ADR injection in Angptl3+/+ mice and persisted throughout the observation period (until week 12). The urine albumin: creatinine ratio showed a significant increase in week 1 (*P* < 0.05), peaked around week 8 (*P* < 0.001) and decreased slightly in week 12 (*P* < 0.01). A similar trend was observed in Angptl3−/− mice injected with ADR. However, the urine albumin: creatinine ratio were significantly lower at every time point (*P* < 0.05) in the Angptl3−/− mice compared to the similarly injected Angptl3+/+ mice (Fig. 1a).

The serum albumin levels in both the Angptl3−/− and Angptl3+/+ mice injected with ADR showed decreasing trends: started to decrease in week 1, and remained decreased significantly throughout the observation period. However, at 4 weeks post ADR injection, the serum albumin levels of the Angptl3+/+ mice were significantly lower than those of the Angptl3−/− mice (*P* < 0.05), and remained significantly lower through week12 (*P* < 0.05) (Fig. 1a).

Renal function, measured as the blood urea nitrogen (BUN) and serum creatinine (Scr) levels, remained stable in the ADR-injected Angptl3+/+ and Angptl3−/− mice during the first 2 weeks but started to decline in week 4. The difference between the treated and control mice became statistically significant in week 8 and continued to be significant for the remainder of the experiment. The BUN and Scr levels were significantly lower in the ADR-injected Angptl3−/− mice compared to than in the ADR-injected Angptl3+/+ mice following ADR injection at 8 and 12 weeks (all *P* < 0.05) (Fig. 1a).

At 3 days post ADR injection, the Angptl3+/+ mice started to look sicker than the Angptl3−/− mice. The Angptl3+/+ mice showed increased hair loss, and their hair appeared dull compared with that of the Angptl3−/− mice. The Angptl3+/+ mice demonstrated reduced activity; compared with the Angptl3−/− mice, the Angptl3+/+ mice maintained a curled body position (data not shown but available from the authors). A higher proportion of the Angptl3+/+ than Angptl3−/− mice died throughout the observation period (Fig. 1b).

**Fig. 2:**
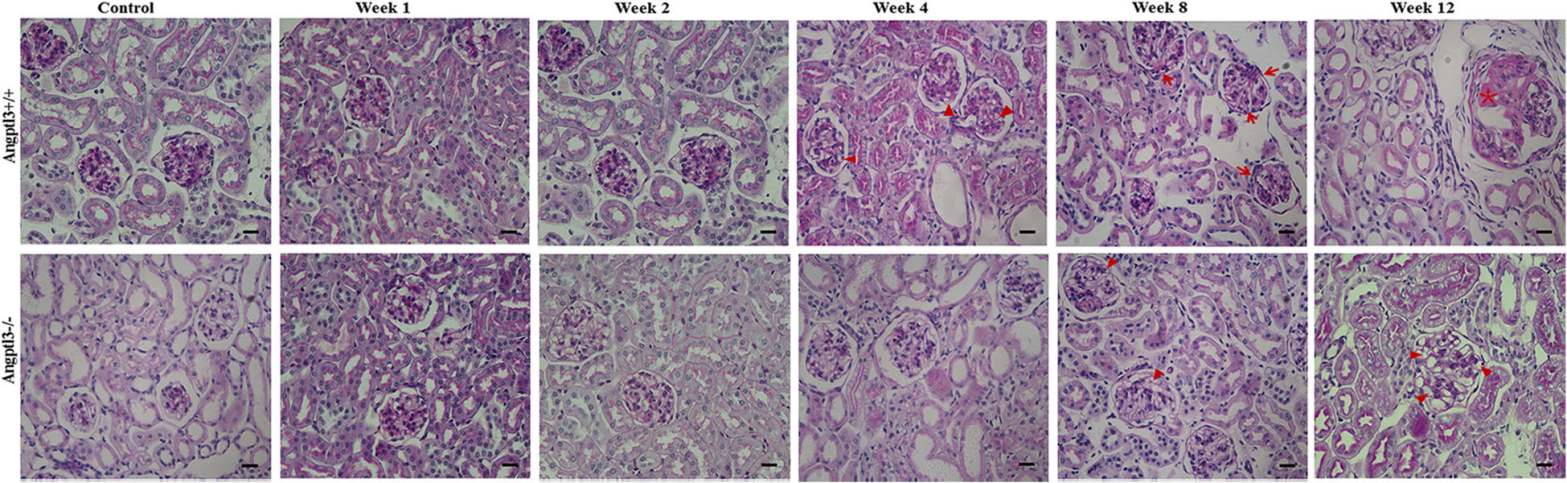
Angptl3 knockout protects renal structure. Representative PAS-stained kidney sections harvested at different time points from Angptl3+/+ and Angptl3−/− mice treated with or without ADR injection (400 ×; scale bars = 15 μm; arrowheads in the image indicate podocyte vacuolization; arrows indicate balloon adhesions; * indicates glomerulosclerosis formation)

Histological examination by light microscopy revealed no evidence of renal injury during the first 2 weeks in either the Angptl3+/+ or Angptl3−/− mice injected with ADR. The Angptl3+/+ mice receiving ADR injection showed podocytes vacuolization in week 4, podocytes vacuolization and balloon adhesions appeared in week 8. Two-thirds (4/6) of the Angptl3+/+ mice manifested typical glomerulosclerosis formation in week 12. In contrast, the ADR-injected Angptl3−/− mice showed occasional podocyte vacuolization in week 8, and glomerulosclerosis was absent in all of the ADR-injected Angptl3−/− mice in week 12 (Fig. 2).

**Fig. 3:**
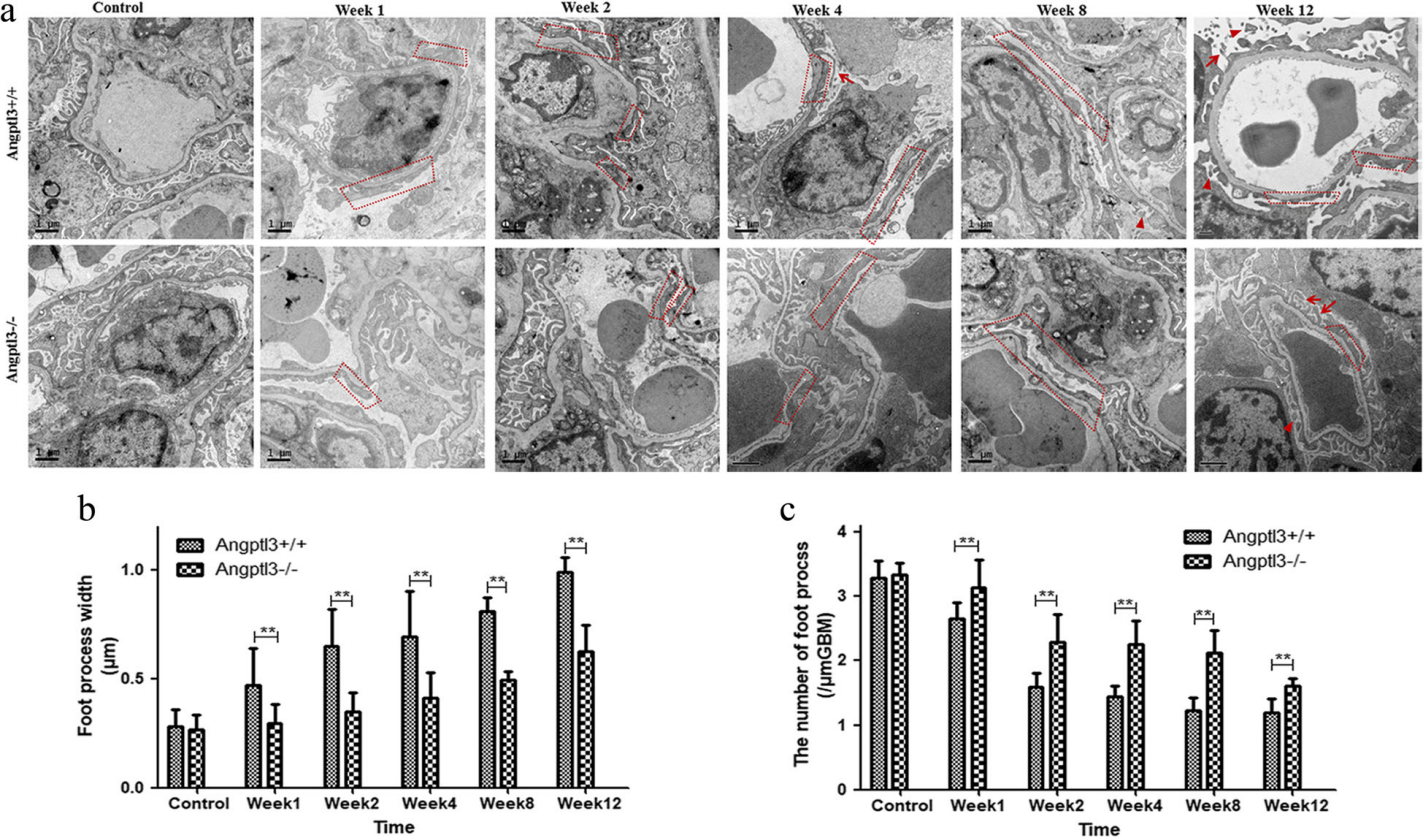
Angptl3 knockout protects the ultrastructure of podocytes. Representative kidney ultrastructure observed with a TEM (**a**) and the quantification of foot process width (**b**) and number (**c**) of Angptl3+/+ and Angptl3−/− mice treated with or without ADR injection (10,000 ×; scale bars = 1 μm; red frames indicate podocyte foot process effacement; arrows indicate podocyte microvillus transformation; arrowheads indicate foot process detachment). Data are shown as the mean ± SEM; * *P* < 0.05 and ** *P* < 0.01

Examination with a TEM showed significant differences in the podocyte lesions, including foot process effacement, microvillus transformation and focal detachment from the basement membrane between the Angptl3+/+ and Angptl3−/− mice with receiving ADR injection from week 1 to week 12. The podocyte lesions progressed more rapidly and were more severe in the Angptl3+/+ mice receiving ADR injection than in the Angptl3−/− mice receiving ADR injection. As observed in week 1, podocyte foot process effacement manifested in Angptl3+/+ mice, while an almost normal podocyte structure was observed in the Angptl3−/− mice. Multifocal podocyte foot process effacement and significant podocyte microvillus transformation was observed in the Angptl3+/+ mice, while fewer podocyte foot process effacement and no microvillus transformation were detected in the Angptl3−/− mice in week 4; Apart from multifocal podocyte foot process effacement and significant podocyte microvillus transformation, focal podocyte foot process detachment from the basement membrane appeared earlier in the Angptl3+/+ mice week8, while, the same pathological lesions were seen in Angptl3−/− mice until week 12 (Fig. 3).

### Angptl3 knockout delays glomerulosclerosis formation in mice with ADR nephropathy by attenuating podocyte loss

**Fig. 4:**
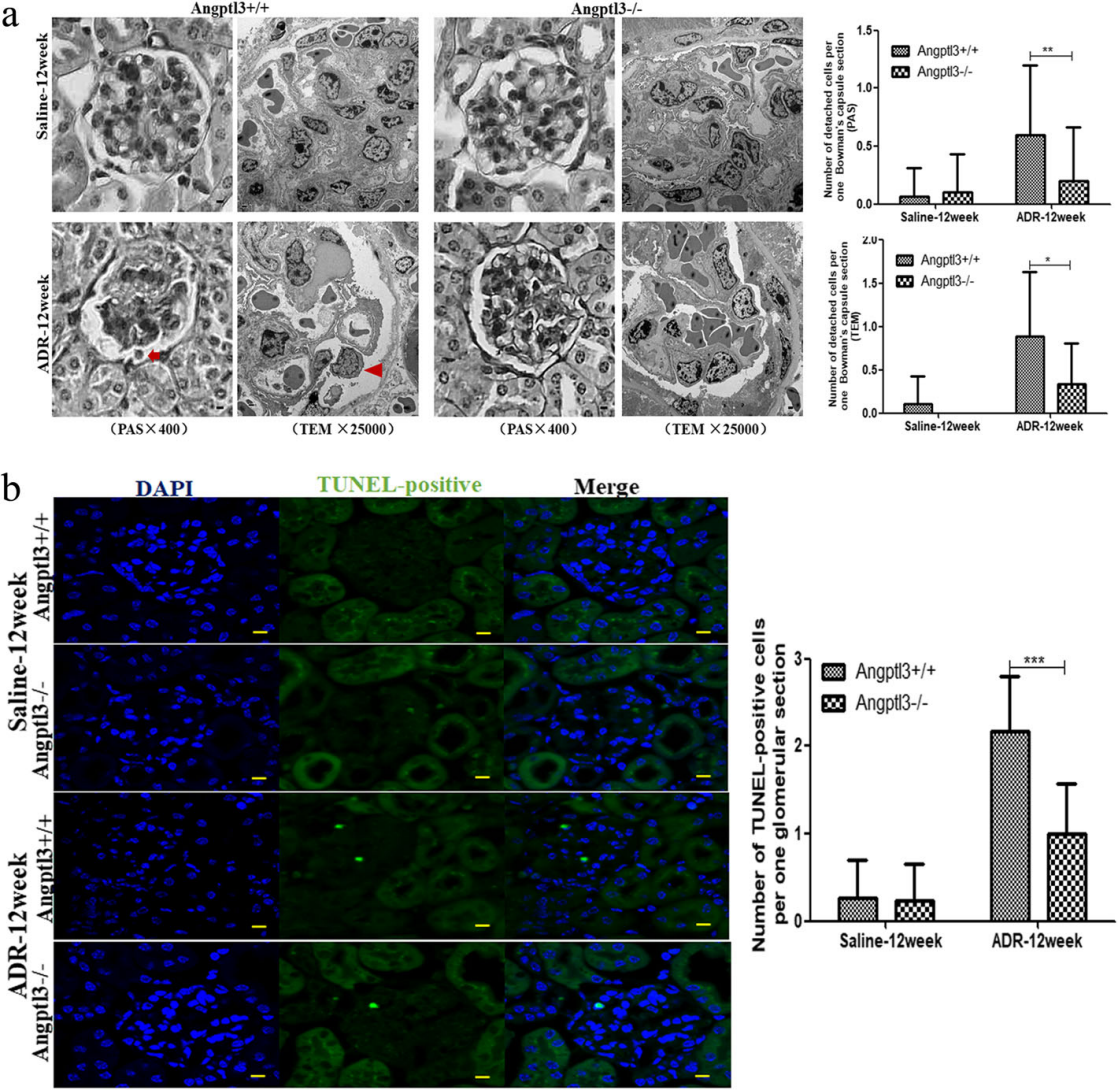
Angptl3 knockout reduces cell loss in renal glomeruli of mice with glomerulosclerosis. **a** Detached cells in Bowman’s capsule of Angptl3+/+ and Angptl3−/− mice treated with ADR injection for 12 weeks. The detached cells in Bowman’s capsule were analyzed under a light microscope and TEM. (PAS staining image with 400 × magnification, scale bars = 5 μm, 30 glomeruli from each mouse, 6 mice per group; transmission electron microscopy image with × 25,000 magnification, scale bars = 1 μm, 1 glomeruli from each mouse, 6 mice per group). **b** Apoptotic cells in glomeruli of Angptl3+/+ and Angptl3−/− mice treated with ADR injection for 12 weeks (630 ×, scale bars = 5 μm, 30 glomeruli from each mouse, 6 mice per group). Data are shown as the mean ± SEM; * *P* < 0.05 and ** *P* < 0.01

Podocyte injury and the subsequent cell loss promote glomerulosclerosis in various glomerular diseases [25, 26]. If cells are lost, then it should be possible to visualize this process by structural analysis. In this partial study, histological sections stained with PAS were examined under a light microscope for detached cells (detached from the glomerular basement membrane [GBM]), and a TEM was used to show rounded cells in Bowman’s capsule. The number of detached cells in Bowman’s capsule was calculated, and the average number of detached cells per randomly chosen glomerular section from the Angptl3+/+ mice injected with ADR for 12 weeks was significantly greater than that of the Angptl3−/− mice undergoing a similar injection protocol (*P* < 0.01, *n* = 30). Analysis with a TEM showed that the average number of detached, rounded cells per randomly chosen glomerular section in the Angptl3+/+ mice injected with ADR for 12 weeks was markedly higher than that of the Angptl3−/− mice undergoing a similar injection protocol (*P* < 0.01, *n* = 6) (Fig. 4a).

Transferase-mediated dUTP nick-end labeling (TUNEL) staining for identifying apoptotic cells in the renal tissue was performed with the different groups. TUNEL-positive cells were not observed in the glomeruli of saline-injected mice. However, after ADR injection for 12 weeks, TUNEL-positive cells were present in the glomeruli of both Angptl3+/+ and Angptl3−/− mice, and the number of TUNEL-positive cells per glomerulus in the Angptl3+/+ mice was greater than that in the Angptl3−/− mice (*P* < 0.05, *n* = 30) (Fig. 4b).

**Fig. 5:**
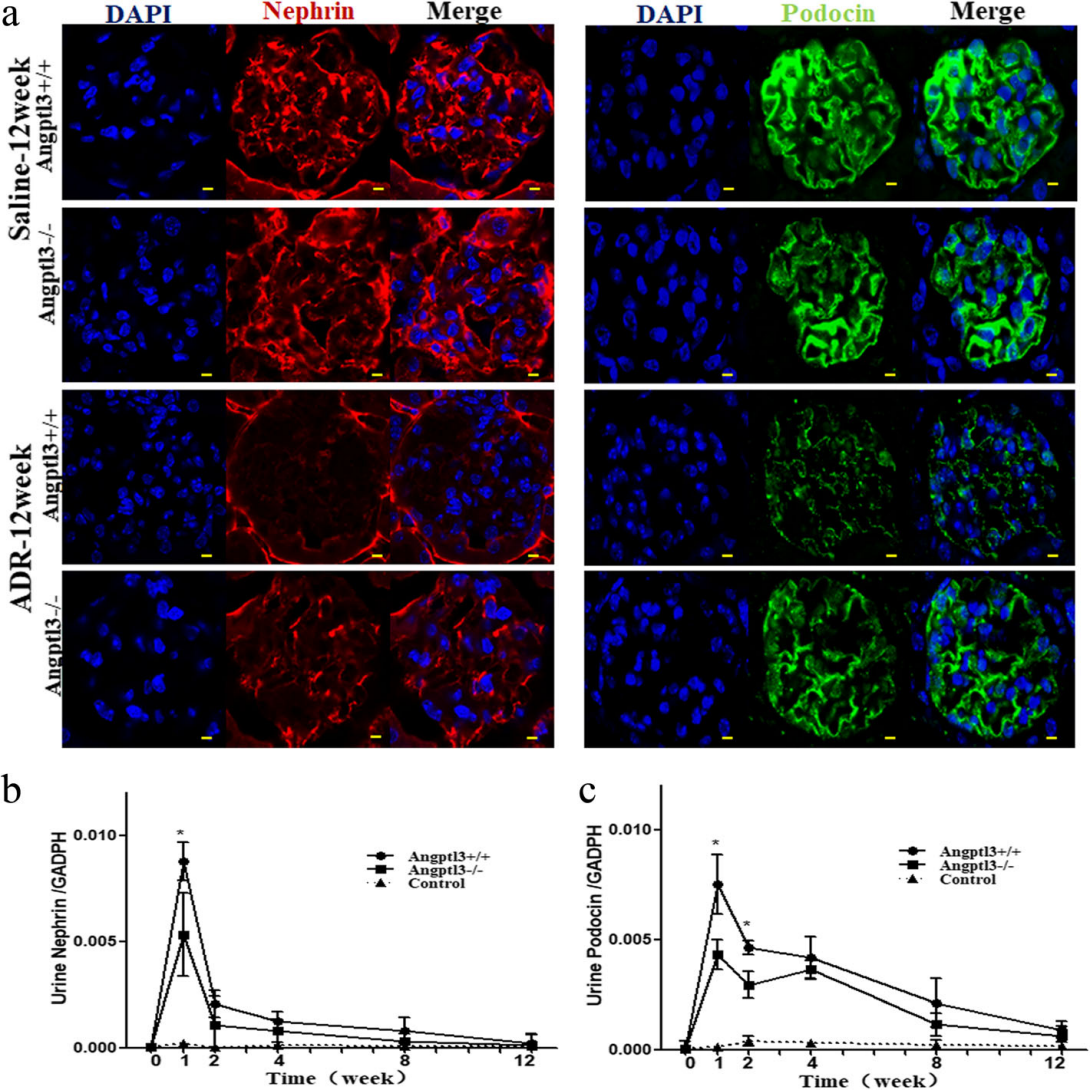
Angptl3 knockout alleviates the loss of podocytes from glomeruli to the urine in the ADR nephropathy mouse model. **a** Podocyte density in glomeruli of Angptl3+/+ and Angptl3−/− mice treated with or without ADR injection for 12 weeks detected by immunofluorescence staining for the podocyte-specific markers nephrin and podocin and confocal microscopy (630 ×, scale bars = 5 μm, *n* = 9). The mRNA levels of the podocyte-specific markers nephrin (**b**) and podocin (**c**) in the urine of Angptl3+/+ and Angptl3−/− mice treated with ADR injection from week 1 to week 12. Data are shown as the mean ± SEM; *n* = 6 per group; * *P* < 0.05

Most of the detached cells present in Bowman’s capsule appeared to be podocytes. To confirm podocyte loss in mice with ADR nephropathy, two podocyte-specific markers, nephrin and podocin, were assessed by immunofluorescence staining and confocal microscopy to detect the podocyte density in glomeruli. The fluorescence intensities of nephrin and podocin were both reduced significantly in glomeruli receiving ADR injection for 12 weeks compared with control glomeruli. Furthermore, the fluorescence intensities of both markers in Angptl3+/+ mice were weaker than those in Angptl3−/− mice (Fig. 5a).

Urine podocyte mRNAs mark the progression of glomerular disease [27]. To examine the hypothesis that podocytes are lost from glomeruli to the urine after ADR injection, positive cells in the urine were analyzed for evidence of podocyte loss using real-time PCR. Primers for nephrin and podocin were used. Nephrin and podocin mRNAs were not detected in mice without ADR injection. Additionally, nephrin and podocin mRNA levels in Angptl3+/+ and Angptl3−/− mice injected with ADR for 12 weeks were also too low to be detected (might be due to detached podocytes undergoing apoptosis). Hence, we detected these mRNAs earlier, 1 week after ADR injection, and positive signals for nephrin and podocin were detected in both Angptl3+/+ and Angptl3−/−mice. Urine podocin mRNA remained elevated throughout the time course before 8 weeks, and returned to normal after 8 weeks of ADR nephropathy progression. In contrast, the nephrin mRNA levels were not correspondingly elevated, as they peaked in week 1 and then decreased, and no further signal was detected in week 12. Both the urine nephrin and podocin mRNA levels in the Angptl3−/− mice were markedly higher than those in the Angptl3+/+ mice in week 1 (*P* < 0.05). The podocin mRNA level in the Angptl3−/− mice was still higher than that in the Angptl3+/+ mice in week 2 (*P* < 0.05), but there was no significant difference in the nephrin mRNA level between the Angptl3+/+ mice and the Angptl3−/− mice in week 2 (*P* > 0.05). After week 2, the nephrin and podocin mRNA levels were comparable between the two groups over the rest of the observed time course (Fig. 5b and c).

### Angptl3 knockout attenuates ADR-induced primary podocyte loss, including detachment and apoptosis, in vitro

**Fig. 6:**
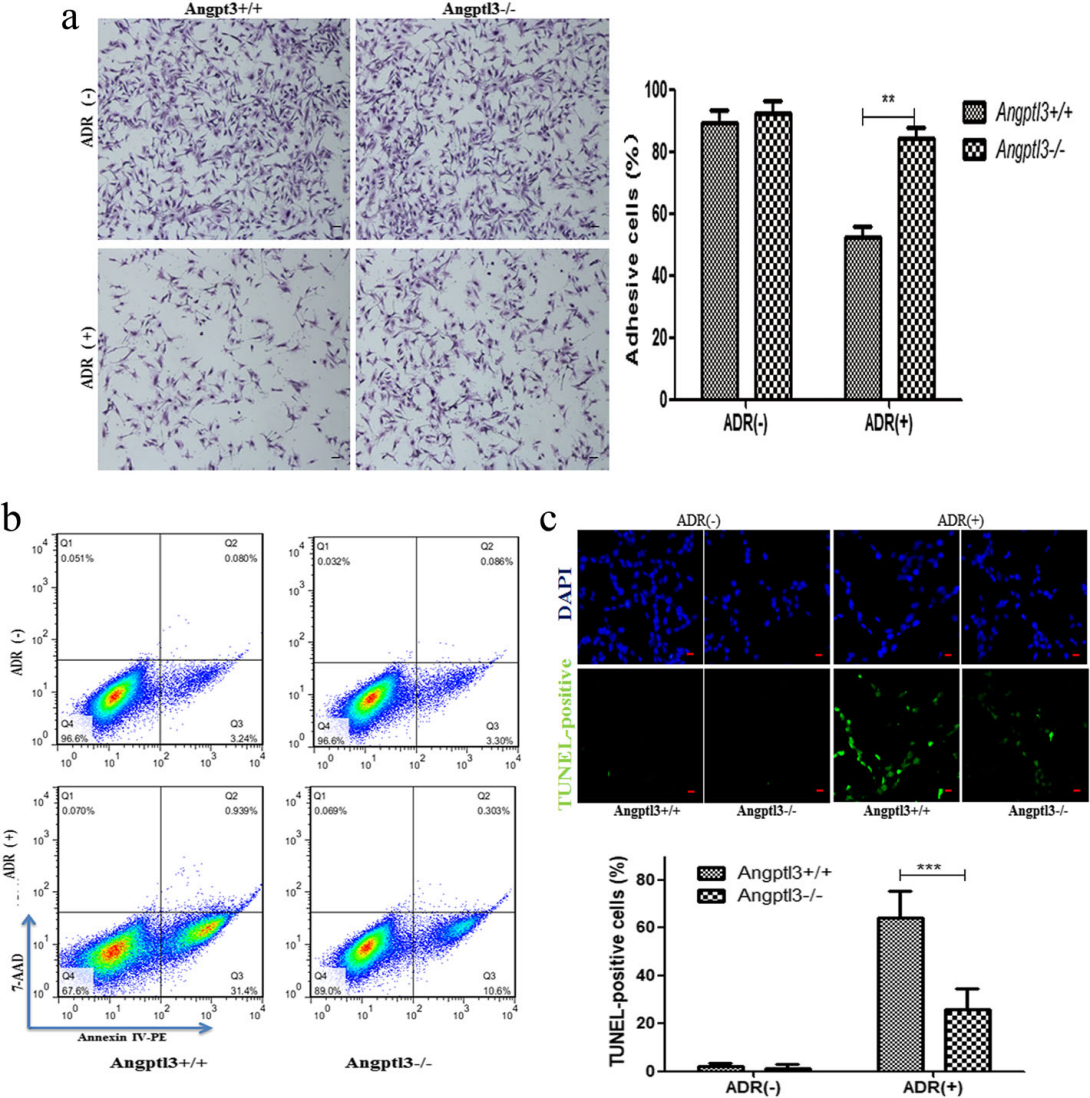
Angptl3 knockout attenuates the ADR-induced primary podocyte loss including detachment and apoptosis, in vitro. Primary podocytes of Angptl3+/+ and Angptl3−/− mice treated with or without ADR attached to the bottom of culture dishes (100 ×, scale bars = 30 μm, *n* = 6), and the rates of cell attachment in the four groups were calculated (**a**). Podocyte apoptosis was analyzed by flow cytometry analysis (**b**) and a TUNEL assay (200 ×, scale bars = 15 μm, *n* = 6) (**c**). Data are shown as the mean ± SEM; *n* = 6 per group; **P < 0.01 and ***P < 0.001; ADR (−): podocytes with PBS treatment; ADR (+): podocytes with ADR treatment. ADR (+): podocytes with ADR treatment

To further explore the mechanism of podocyte loss, primary podocytes from Angptl3+/+ and Angptl3−/− mice were cultured, and ADR was used to induce podocyte loss, including detachment and apoptosis. The proportions of attached podocytes in the Angptl3+/+ and Angptl3−/− groups without ADR treatment were similar. However, after ADR treatment, a significant difference was noted in the proportion of attached podocytes between the Angptl3+/+ and Angptl3−/− groups; the proportion of attached podocytes in the former group was markedly lower than that in the latter group (Fig. 6a).

Podocyte apoptosis was detected by flow cytometry analysis and TUNEL labeling under the same experimental conditions as those used for the detachment assay. Apoptosis was rarely observed in either the Angptl3+/+ or Angptl3−/− group without ADR treatment. After ADR treatment, the rate of apoptosis detected by flow cytometry in the Angptl3+/+ and Angptl3−/− groups were higher than those in the control groups, and the apoptosis rate in the Angptl3−/− group was significantly lower than that in the Angptl3+/+ group (*P* < 0.01) (Fig. 6b). A similar and more sensitive finding was obtained using the TUNEL assay (*P* < 0.001) (Fig. 6c).

Our previous study verified that integrin α3β1, integrin-linked kinase (ILK), and p53 are necessary for Angptl3 to affect PAN-induced podocyte loss [18]. In the current study, the levels of integrin α3β1and phosphorylation of integrin β1, ILK and p53 were detected to re-elucidate the molecular mechanism underlying the amelioration of podocyte loss and glomerulosclerosis in the context of Angptl3 knockout.

The expression of integrinα3 in Angptl3−/− podocytes treated with ADR was weaker than that in control podocytes but stronger than that in Angptl3+/+ podocytes treated with ADR. In comparison to that in the control groups, the expression of integrinβ1, phospho-integrin β1 and ILK in the Angptl3−/− podocyte group treated with ADR were not altered. However, in the Angptl3+/+ podocytes treated with ADR, the expression of integrinβ1 decreased, and phospho-integrin β1 and ILK expression increased (Additional file 2: Figure S2a). No p53 protein was observed in the untreated Angptl3+/+ or Angptl3−/− podocytes, but after ADR treatment, p53 protein expression was observed in the two sets of podocytes, and the level in the Angptl3−/− group was lower than that in the Angptl3+/+ group (Additional file 2: Figure S2b). Taken together, the protein analyses confirmed that following ADR treatment, lower expression of integrinα3 β1 and p53 and higher expression of phospho-integrin β1 and ILK were observed in the Angptl3+/+ podocytes than that in the Angptl3−/− podocytes.

## Discussion

The ADR-induced nephropathy mouse model is ideal for clarifying the underlying mechanisms of the response of podocytes to injury in kidney disease [20]. The successful establishment of the ADR nephropathy mouse model is closely related to many factors, such as the mouse susceptibility to ADR, the dosing of ADR, the method of administration, and the frequency and interval time of the method. In the current study, we successfully established an ADR nephropathy mice model on a B6; 129S5 genetic background with ADR (25 mg/kg) administered intravenously once via tail vein injection and monitored for long-term follow-up. Our ADR nephropathy mice model on a B6; 129S5 genetic background was manifested with minimal change disease in the early stage (1 week after ADR injection) and glomerulosclerosis formation in the end stage (12 week post ADR injection), which further facilitated the in vivo study of the pathophysiology and treatment of chronic proteinuric renal disease.

Angptl3 knockout was observed to play a crucial protective role (attenuated proteinuria and hypoproteinemia, and improved renal structure and function as well as the general condition and survival of mice) in the whole process of ADR nephropathy. In the early stage, the Angptl3 knockout greatly attenuated proteinuria and maintained the integrity of the podocyte foot process, which was consistent with the observations of our previous study [19]. In the end stage, Angptl3 knockout effectively delayed glomerulosclerosis formation. The present results suggested that antagonists or inhibitors such as small molecule drugs or antibodies targeting Angptl3 might be specific and effective candidate therapeutic approaches for podocyte injury and proteinuria in nephropathy, and if those therapeutic approaches were used in treatments, continuously maintaining a low level of Angptl3 (long-term medication) would have better effectiveness.

The loss of cells in glomeruli beyond a critical level results in widespread glomerulosclerosis leading to the progressive loss of renal function culminating in end-stage of kidney disease [1, 2]. The most important of the lost cells are podocytes, and if progressive podocyte loss is allowed to occur over time, then this loss is associated with progressive glomerulosclerosis [2, 3]. In this partial study, we found that Angptl3 knockout reduced the number of detached cells in glomerular Bowman’s capsule and attenuated the number of apoptotic cells in the renal tissue of mice with end-stage ADR nephropathy. We demonstrated that Angptl3 knockout attenuated podocyte loss (alleviated the decrease in podocyte density in the renal tissue and reduced the urine podocyte mRNA levels) in mice with end-stage ADR nephropathy, and we suggested that most of the lost cells were podocytes. These findings revealed that Angptl3 knockout could ameliorate glomerulosclerosis formation in mice with ADR nephropathy by attenuating podocyte loss.

Podocyte detachment and apoptosis are two risk factors that cause podocyte loss [21, 22]. Podocytes adhere to the GBM principally via integrinα3β1 [28], and ILK plays a key role in integrinα3β1-mediated podocyte adhesion [29]. The tumor suppressor protein p53 is involved in the crucial processes of podocyte apoptosis [30]. Our previous study confirmed that Angptl3 is a novel factor that is involved in the PAN-induced podocyte loss by affecting detachment and apoptosis in vitro. Knockdown of Angptl3 by small interfering RNA (siRNA) markedly ameliorated podocyte loss, and the observed effects were partially correlated with the altered integrinα3β1, ILK and p53 [18]. Culturing primary podocytes seemed to be a bridge between cell lines and in vivo growth, and produces cells with characteristics closer to the biological characteristics of podocytes in vivo [31]. In the current study, we cultured primary podocytes from Angptl3+/+ and Angptl3−/− mice in vitro to further explore whether Angptl3 knockout ameliorates podocyte loss. We demonstrated that Angptl3 knockout effectively rescued primary podocytes from detachment and apoptosis induced by ADR, and Integrin α3β1, ILK, and p53 were altered markedly in the observed process. These results powerfully support our previous viewpoint that lowering Angptl3 expression or knockout Angptl3 may ameliorate the loss of podocytes via reductions in podocyte detachment and apoptosis in vitro, and the observed effects partially correlated with the alterations in integrinα3β1, ILK and p53. Notably, comparing with our previous study [18], this study showed that Angptl3 knockout tended to protect podocyte loss more effectively than the Angptl3 lower by siRNA, which might invite speculation regarding a “dose-dependent effect” of Angptl3 on podocyte loss. Generally, the lowering the expression of Angptl3 resulted in a better effect on renal protection. However, the exact phenomenon and mechanism need to be further studied.

Recent years have seen dramatic advances in the understanding of the roles of Angptl3 in podocyte injury. Our findings provide more information on the identification of potential and specific therapeutics targeting Angptl3 in podocyte injury and proteinuria. However, our studies are limited. Firstly, the PAN model may also be used as a model of glomerulosclerosis or minimal change disease based on the amount of PAN injected [32]. Our results need to be confirmed in the PAN model. Second, we previously demonstrated that Angptl3 is involved in the development of proteinuria by triggering integrin β3 and the downstream FAK/PI3K signaling pathway in podocytes. Either decreasing ANGPTL3 expression or interfering with the ANGPTL3-integrin β3 interaction might be beneficial for protecting and decreasing proteinuria [17, 19]. However, in the current study, our finding indicated that Angptl3 knockout could attenuate proteinuria, but could not alleviate proteinuria completely. The possible cause may be that even though we inhibited or deleted Angptl3, integrin β3 might have been activated by other factors, such as urokinase receptor (uPAR), to promote the effacement of podocyte foot processes [33] and produced proteinuria [34]. The results suggested that candidate therapeutic approaches targeting on Angptl3 should be combined with other therapeutic options to produce potent therapeutic effects on proteinuria. Finally, ADR nephropathy is a highly reproducible model of renal injury. It is also a “robust” model in that the degree of tissue injury is severe while being associated with acceptable mortality and morbidity [20]. In the current study, we observed that in week 1 post ADR injection, a small number of mice including both Angptl3+/+ and Angptl3−/− mice started to die, although we did not observe the heart function and pathology of these dead mice, the putative cardiotoxicity of doxorubicin might be then main causes of death [35]. In addition, we observed the liver function and structure of weak mice (close to death) and found that their blood alanine aminotransferase (ALT) levels were abnormally high and that their liver structure showed pathologic vacuoles (data not shown but available from authors). Therefore, we speculated that the cause of death in mice receiving ADR injection was not only cardiac dysfunction, but also abnormal liver function. Previous studies suggest Angptl3 is mainly produced by the liver [4] and associated with liver health [36]. A higher proportion of Angptl3+/+ mice than Angptl3−/− mice died throughout our observation period, which might indicate that the Angptl3 knockout also played an important role in hepatic protection. In-depth studies regarding the hepatic protection mediated by Angptl3 knockout are also necessary.

## Conclusions

Our study is the first to demonstrate that in addition to serving in renal- protective roles in the early stage of nephropathy, the Angptl3 knockout may play a crucial role in ameliorating glomerulosclerosis by attenuating podocyte loss through rescuing podocytes from detachment and apoptosis in ADR nephropathy. This revelation will help to deepen the understanding of the mechanisms of glomerulosclerosis and podocyte loss, and may indicate that Angptl3 is an attractive therapeutic target in podocyte injury during the occurrence and progression of nephropathy.

## Additional files

Additional file 1: Supplementary methods. (DOCX 20 kb)

Additional file 2: **Figure S1.** Mice model establishment and cellular outgrowth from glomeruli, as well as the activity and purity of primary podocytes. (a) A schematic representation shows the mouse model experimental procedure. (b) Glomeruli were isolated, and no cellular outgrowth was observed in this attached glomerulus after 0.5 days in culture; capsulated glomeruli (containing the glomerular tuft, Bowman’s membrane and PEC) are indicated by arrowheads, and decapsulated glomeruli (containing the glomerular tuft only) are indicated by large arrows. The first evidence of cells (represented by small arrows) emerging from the glomerulus was found after 4 days in culture; the glomerulus was surrounded by a colony of cells, and the putative podocytes were all uniform in shape and size. (c) An MTT assay revealed that the cell number of the p1 podocytes increased between 3 days and 7 days, and then, a decrease in cell number was observed between day 8 d and day 10. (d) Immunostaining for the podocyte-specific proteins nephrin (red) and wt1 (red) and (e) FACS analysis (nephrin) were used to identify the purity of the primary podocytes, Scale bars = 15 μm. P0–0d: Passage 0, day 0; P0–0.5d: Passage 0, after half a day; P0-4d: Passage 0, 4th day; P1-7d: Passage 1, 7th day. **Figure S2.** Angptl3 knockout influences integrinα3β1, ILK and p53 in primary podocytes with ADR treatment. Western blot analyses were performed to measure the expression of integrin α3 and integrin β1 and the phosphorylation of integrin β1, integrin-linked kinase (ILK) and p53 in primary podocytes from Angptl3+/+ and Angptl3−/− mice treated with or without ADR. The relative levels of integrinα3, total integrin β1, phospho-integrinβ1, ILK **(a)** and p53 **(b)** were determined. Data are shown as the mean ± SEM; *n* = 6 per group; *P < 0.05 and **P < 0.01; ADR (−): podocytes with PBS treatment. (ZIP 10021 kb)

## Abbreviations

Angptl3: Angiopoietin-like3
BUN: Blood urea nitrogen
CKD: Chronic kidney disease
ESRD: End stage renal disease
MMRRC: Mutant Mouse Regional Resource Centers
mRNAs: Messenger ribonucleic acids
PAN: Puromycin aminonucleoside
PAN: Puromycin aminonucleoside
PAS: Periodic acid–Schiff
PCR: Polymerase chain reaction
Scr: Serum creatinine
siRNA: Small interfering RNA
TEM: Transmission electron microscope
TUNEL: Transferase-mediated dUTP nick-end labeling
uPAR: Urokinase receptor
