# Supplementary material for: Angiopoietin-like-3 knockout protects against glomerulosclerosis in murine adriamycin-induced nephropathy by attenuating podocyte loss
Source: BMC Nephrol. 2019 May 24;20:185. doi: 10.1186/s12882-019-1383-1 (PMC6533758; doi:10.1186/s12882-019-1383-1)
Supplement: Supplementary file 1 — Supplementary methods. (DOCX 20 kb) [file 12882_2019_1383_MOESM1_ESM.docx]

**Urine Protein Excretion Assay**

Urinary albumin and creatinine was tested by ELISA kit (Cloud-Clone Corp, Katy, USA). The albumin/creatinine ratio was calculated. Student’s t test was used for statistical analysis. P < 0.05 was considered to be statistically significant.

**Serum biochemistry analysis**

Blood was centrifuged at 12 000×g for 15min, and the supernatant was tested by automatic biochemical analytical instruments (Vetscan vs2 comprehensive diagnostic). Analysis of variance (ANOVA) was used for the statistical analysis. P < 0.05 was considered to be statistically significant.

**Periodic acid–schiff (PAS) histostain**

Samples were dewaxed and rehydrated through descending graded alcohols to PBS, pH 7.4, and were then treated with 1% periodic acid for 10 min and washed in water, followed by Schiff reagent for 10 min. They were then washed for several minutes in tap water to bring out the color of Schiff. The nuclei were stained with Mayer’s hematoxylin for 2 min, were washed in tap water, were immersed in “bluing solution” for 2 minutes, and were then returned to water. Tissue sections were dehydrated in graded ethanol, cleared in xylene, and mounted in sections.

**Transmission electron microscopy**

The renal cortices were cut (1 mm^3^) and fixed in 2.5% glutaraldehyde overnight (4°C), post fixed (24°C) in 1 % osmium tetroxide in the same phosphate buffer and dehydrated in increasing concentrations of alcohol and embedded in araldite according to routine procedures. Sections (approximately 70 nm thickness) were contrast stained with uranyl acetate and examined under transmission electron microscope (Philips CM120).

**Immunofluorescence labeling**

Cryosections (4 μm) were ﬁxed in ice-cold acetone and were then stained with the primary antibodies, nephrin mouse monoclonal antibody, podocin rabbit polyclonal antibody (Abcam, Cambridge, UK). The sections were then incubated with either FITC-conjugated or rhodamine-conjugated secondary antibodies. Nuclei were stained with 4′6′-diamidino-2-phenylindole dihydrochloride (DAPI) for 20 min at room temperature. The sections were examined by fluorescence microscopy and LSM 710 laser confocal microscopy (Carl Zeiss, Thornwood, USA) at a magnification of 630× and merged images were generated using Adobe Photoshop CS software. The ﬂuorescent images were collected during a single rapid scan with identical parameters for all samples.

**MTT assay**

Primary podocytes activity was assessed by MTT assay (Promega, Madison, WI) as published previously. Briefly, cells were plated into a 96-well plate; allowed to adhere overnight; and cultured for 1 to 10 d. MTT was added at a final concentration of 0.5 mg/mL to the wells, cells were incubated for 4 hours at 37℃, and the purple formazan crystals were solubilized in dimethyl sulfoxide (DMSO). Optical density was measured with a microplate reader (Benchmark) (Bio-Rad, Munich, Germany) at a reference wavelength of 570 nm. Six wells were measured for each time point in each experiment. Data are presented as means ± SEM of three experiments.

**Detachment Assay**

The detachment of podocytes was assayed according to our previous study[ ^1^5,18]. All cells were cultured on six-well plates under nonpermissive conditions for 14 days prior to the experiments, fields of cells were marked, and cell numbers per field were counted to establish a baseline number. The mean numbers of different fields in three independent sets in this experiment were determined.

**Detection of Apoptosis**

Both PE-Annexin-V detection of apoptosis and deoxynucleotidyl transferase-mediated dUTP nick-end labeling (TUNEL) assay were used to detect apoptosis. Methods were performed using PE-Annexin-V Apoptosis Detection Kit (BD Biosciences, San Diego, USA) and In Situ Cell Death Detection kit (Roche, Mannheim, Germany) following the manufacturer’s protocol. Overall, 6000 cells per experiment were resuspended with PE-Annexin-V and 7-aminoactinomycin D and analyzed using flow cytometry analysis. TUNEL staining was examined under fluorescence microscope (Olympus, Japan), for each group in given experiments, at least 300 randomly chosen cells were determined for the quantification [18].

**Western blot**

Western blotting was performed according to the standard procedures. An equal amount of each protein lysate was loaded onto 8% or 10% sodium dodecyl sulfatepolyacrylamide gel electrophoresis gels and blotted onto polyvinylidene fluoride membranes. Samples were blocked in Tris-buffered saline Tween 20 (20 mM Tris-HCl, pH 7.4 and 0.05% Tween 20) with 5% non-fat dry milk. The membranes were incubated with primary antibodies, mouse monoclonal antibody (mAb) to glyceraldehyde-phosphate dehydrogenase (GAPDH), integrin alpha 3, total integrin beta 1, phospho-integrin beta 1 (phospho T789) and ILK(Abcam, Cambridge, UK); p53 mouse mAb (Cell Signaling Technology, Beverly, USA) at appropriate dilutions overnight at 4°C and horseradish peroxidase-linked secondary antibody (at a dilution of 1:2000) (Santa Cruz, USA) for 1 hour at room temperature. The results were visualized by fluorography using the Tanon gel imaging system (Tanon, Shanghai, China).
