# Supplementary figures and images for: Angiopoietin-like-3 knockout protects against glomerulosclerosis in murine adriamycin-induced nephropathy by attenuating podocyte loss
Source: BMC Nephrol. 2019 May 24;20:185. doi: 10.1186/s12882-019-1383-1 (PMC6533758; doi:10.1186/s12882-019-1383-1)

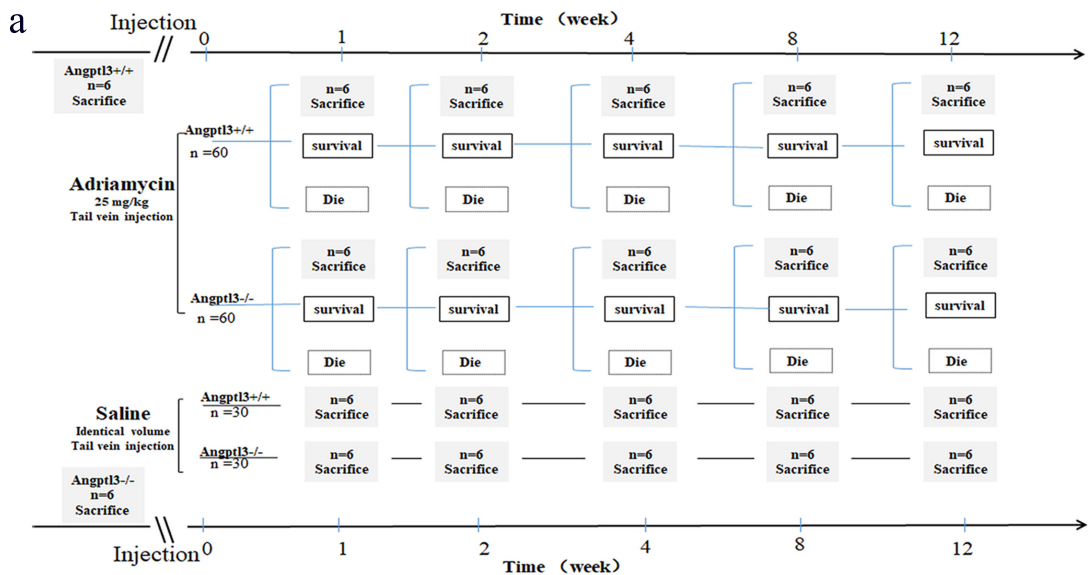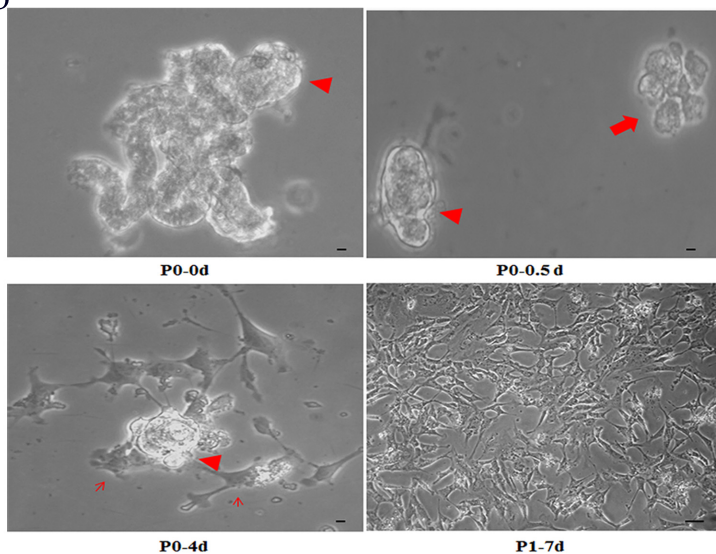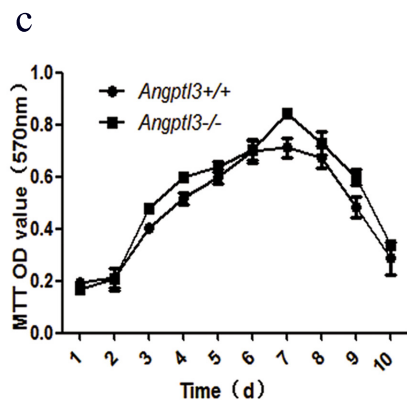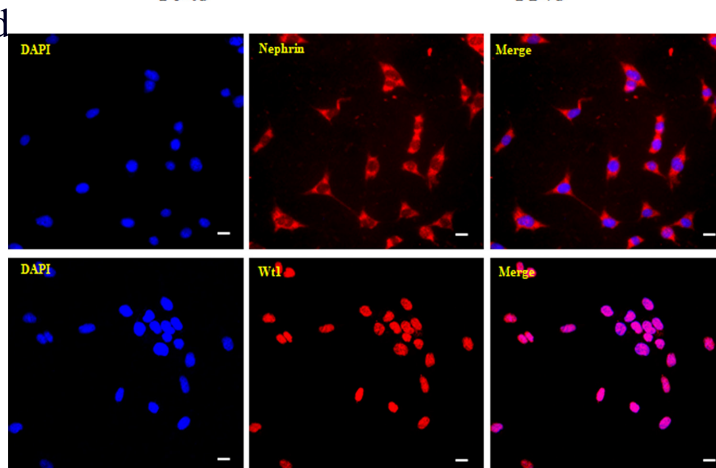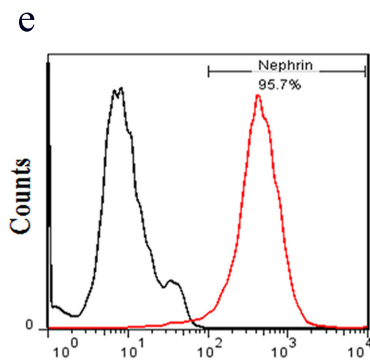

Supplement: Supplementary file 2 — Figure S1. Mice model establishment and cellular outgrowth from glomeruli, as well as the activity and purity of primary podocytes. (a) A schematic representation shows the mouse model experimental procedure. (b) Glomeruli were isolated, and no cellular outgrowth was observed in this attached glomerulus after 0.5 days in culture; capsulated glomeruli (containing the glomerular tuft, Bowman’s membrane and PEC) are indicated by arrowheads, and decapsulated glomeruli (containing the glomerular tuft only) are indicated by large arrows. The first evidence of cells (represented by small arrows) emerging from the glomerulus was found after 4 days in culture; the glomerulus was surrounded by a colony of cells, and the putative podocytes were all uniform in shape and size. (c) An MTT assay revealed that the cell number of the p1 podocytes increased between 3 days and 7 days, and then, a decrease in cell number was observed between day 8 d and day 10. (d) Immunostaining for the podocyte-specific proteins nephrin (red) and wt1 (red) and (e) FACS analysis (nephrin) were used to identify the purity of the primary podocytes, Scale bars = 15 μm. P0–0d: Passage 0, day 0; P0–0.5d: Passage 0, after half a day; P0-4d: Passage 0, 4th day; P1-7d: Passage 1, 7th day. Figure S2. Angptl3 knockout influences integrinα3β1, ILK and p53 in primary podocytes with ADR treatment. Western blot analyses were performed to measure the expression of integrin α3 and integrin β1 and the phosphorylation of integrin β1, integrin-linked kinase (ILK) and p53 in primary podocytes from Angptl3+/+ and Angptl3−/− mice treated with or without ADR. The relative levels of integrinα3, total integrin β1, phospho-integrinβ1, ILK (a) and p53 (b) were determined. Data are shown as the mean ± SEM; n = 6 per group; *P < 0.05 and **P < 0.01; ADR (−): podocytes with PBS treatment. (ZIP 10021 kb) [file 12882_2019_1383_MOESM2_ESM.zip › Supplementary Figure1R3.pdf]

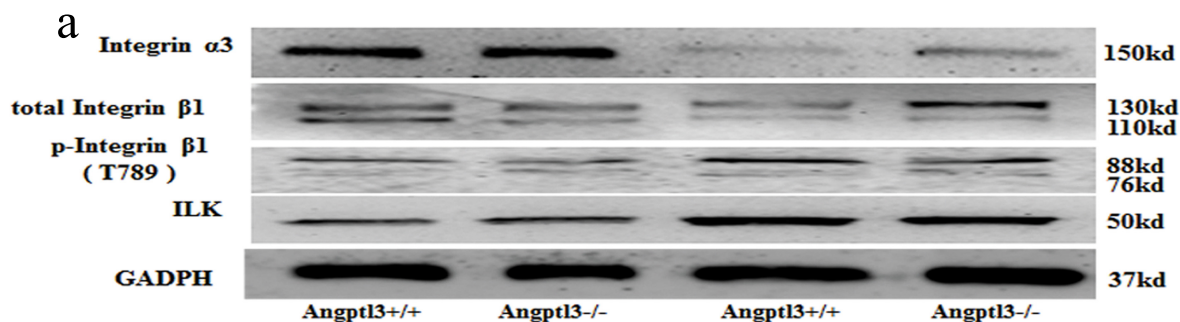

**ADR(-)**

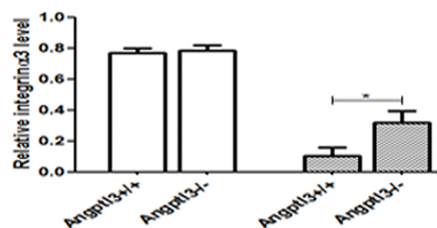

**ADR(+)**

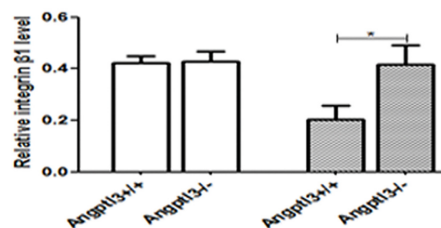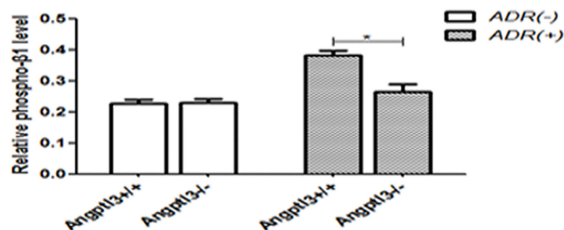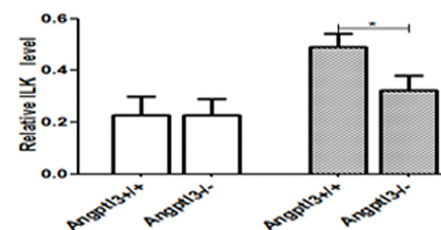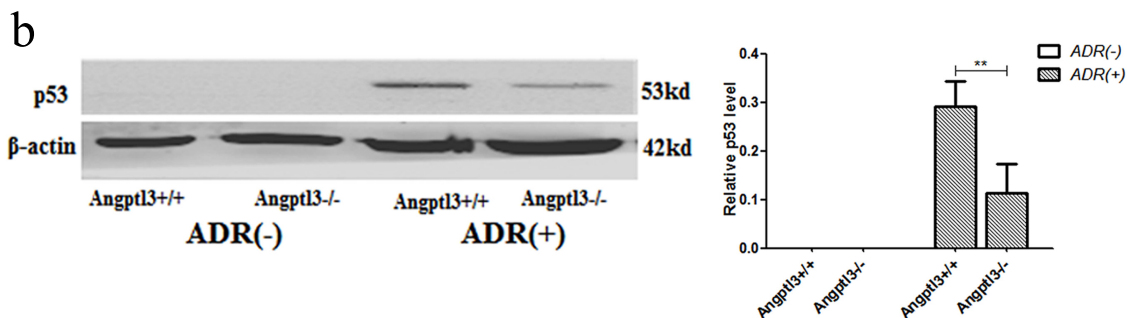

Supplement: Supplementary file 2 — Figure S1. Mice model establishment and cellular outgrowth from glomeruli, as well as the activity and purity of primary podocytes. (a) A schematic representation shows the mouse model experimental procedure. (b) Glomeruli were isolated, and no cellular outgrowth was observed in this attached glomerulus after 0.5 days in culture; capsulated glomeruli (containing the glomerular tuft, Bowman’s membrane and PEC) are indicated by arrowheads, and decapsulated glomeruli (containing the glomerular tuft only) are indicated by large arrows. The first evidence of cells (represented by small arrows) emerging from the glomerulus was found after 4 days in culture; the glomerulus was surrounded by a colony of cells, and the putative podocytes were all uniform in shape and size. (c) An MTT assay revealed that the cell number of the p1 podocytes increased between 3 days and 7 days, and then, a decrease in cell number was observed between day 8 d and day 10. (d) Immunostaining for the podocyte-specific proteins nephrin (red) and wt1 (red) and (e) FACS analysis (nephrin) were used to identify the purity of the primary podocytes, Scale bars = 15 μm. P0–0d: Passage 0, day 0; P0–0.5d: Passage 0, after half a day; P0-4d: Passage 0, 4th day; P1-7d: Passage 1, 7th day. Figure S2. Angptl3 knockout influences integrinα3β1, ILK and p53 in primary podocytes with ADR treatment. Western blot analyses were performed to measure the expression of integrin α3 and integrin β1 and the phosphorylation of integrin β1, integrin-linked kinase (ILK) and p53 in primary podocytes from Angptl3+/+ and Angptl3−/− mice treated with or without ADR. The relative levels of integrinα3, total integrin β1, phospho-integrinβ1, ILK (a) and p53 (b) were determined. Data are shown as the mean ± SEM; n = 6 per group; *P < 0.05 and **P < 0.01; ADR (−): podocytes with PBS treatment. (ZIP 10021 kb) [file 12882_2019_1383_MOESM2_ESM.zip › Supplementary Figure2R3.pdf]
